# Evaluation of Active Heat Sinks Design under Forced Convection—Effect of Geometric and Boundary Parameters

**DOI:** 10.3390/ma14082041

**Published:** 2021-04-18

**Authors:** Eva C. Silva, Álvaro M. Sampaio, António J. Pontes

**Affiliations:** 1 IPC—Institute of Polymers and Composites, Department of Polymer Engineering, Campus de Azurém, University of Minho, 4800-058 Guimarães, Portugal; amsampaio@dep.uminho.pt (Á.M.S.); pontes@dep.uminho.pt (A.J.P.); 2 DONE Lab—Advanced Manufacturing of Polymers and Tools, Campus de Azurém, University of Minho, 4800-058 Guimarães, Portugal; 3 Lab2PT, School of Architecture, Campus de Azurém, University of Minho, 4800-058 Guimarães, Portugal

**Keywords:** heat sink, computational fluid dynamics, simulation, ANSYS Fluent, additive manufacturing, lattice structures, design of experiments

## Abstract

This study shows the performance of heat sinks (HS) with different designs under forced convection, varying geometric and boundary parameters, via computational fluid dynamics simulations. Initially, a complete and detailed analysis of the thermal performance of various conventional HS designs was taken. Afterwards, HS designs were modified following some additive manufacturing approaches. The HS performance was compared by measuring their temperatures and pressure drop after 15 s. Smaller diameters/thicknesses and larger fins/pins spacing provided better results. For fins HS, the use of radial fins, with an inverted trapezoidal shape and with larger holes was advantageous. Regarding pins HS, the best option contemplated circular pins in combination with frontal holes in their structure. Additionally, lattice HS, only possible to be produced by additive manufacturing, was also studied. Lower temperatures were obtained with a hexagon unit cell. Lastly, a comparison between the best HS in each category showed a lower thermal resistance for lattice HS. Despite the increase of at least 38% in pressure drop, a consequence of its frontal area, the temperature was 26% and 56% lower when compared to conventional pins and fins HS, respectively, and 9% and 28% lower when compared to the best pins and best fins of this study.

## 1. Introduction

All electronic devices dissipate heat during their operation. By providing heat dissipation, a heat sink prevents overheating and plays an imperative role in temperature regulation. Through extended surfaces, heat sinks increase heat dissipation from a heat source to the surroundings, providing low thermal resistance (Equation (1)) and a low-pressure loss path between them [1]. They can be divided into two main categories: active and passive cooling techniques. The use of natural techniques is known as passive thermal management while forced heat dissipation, e.g., by cooling fans, improving heat transfer, is referred to as active thermal management [2,3,4,5]. The main advantages of passive cooling techniques are their simplicity and lower cost of operation. However, the associated heat transfer coefficient (*h*) is low [6]. Forced convection with cooling fans is a process frequently found in a variety of electronic products ranging from personal computers to avionics control systems [7]. Airflow speed is actively increased, enhancing heat transfer [6]. The most typical material for heat sinks is aluminium, offering a good balance between weight, cost, and thermal properties [8,9,10,11].

Nowadays, as electronic components continue to dissipate more heat and, with new developments, are getting more compact, their cooling techniques must also be improved [7]. Due to its geometric freedom and the capability to build complex internal structures and with high total area to volume ratio, additive manufacturing can be a useful way to produce heat sinks that match or outperform the thermal performance of traditional aluminium heat sinks [12]. Chinthavali et al. [13] produced the first heat sink for electronic components by additive manufacturing using powder-bed fusion (PBF) equipment. The toughness of the heat sink produced with the additive manufacturing aluminium alloy was similar to the strength of the heat sink produced by conventional methods, but the thermal performance was lower for lower temperatures. Later, Syed-Khaja et al. [14] used PBF to fabricate a heat sink design that showed key enabling advantages such as the reduction in volume, weight, and chip temperatures. To date, several other studies have emerged regarding heat sinks produced by additive manufacturing [5,15,16,17,18,19,20].

This study aims to evaluate the performance of different heat sinks. In the first stage, the influence of different geometric and boundary parameters on the performance of conventional fins/pins heat sinks will be evaluated. Based on those results, the design of the heat sinks will be changed considering some additive manufacturing approaches and compared with lattice heat sinks, which are complex structures only possible to be produced by additive manufacturing. Finally, the best heat sink design for forced convection environments, among the studied, will be revealed.

Heat sinks performance was evaluated considering their thermal resistance (Equation (1)), which is one of the main indicators and should be as low as possible. It is expressed as:

(1) $$R=\frac{\Delta T}{Q_{heat}}$$

where ∆*T* is the difference between the minimum temperature of the heat sink and the fluid temperature at the inlet and *Q_heat_* is the total heat applied at the base of the heat sink, given by multiplying the heat flux by the base area [21,22].

In this work, both the heat source and air flow inlet velocity are constant parameters and were defined at the base of the heat sink and the beginning of the wind tunnel, respectively. The performance of a heat sink design was evaluated by measuring their temperature after 15 s of applied heat and air flow (directly correlated with the thermal resistance according to Equation (3)). Besides the temperature control, the air pressure drop after 15 s was used as an auxiliary control metric to compare studies where the temperature differential was residual or an anormal air pressure was identified.

### 1.1. Conventional Heat Sinks Design and Topology

Heat sink design is the most important variable for better performance. It minimizes thermal resistance by expanding the surface area available for heat transfer while ensuring that the air flows through the heat sink [3].

Choosing the type of heat sink (pins, fins, or blades) is an ambiguous task. Under forced convection, Wong et al. [23] defended that fins are the best choice (among fins, blades, and circular pins), while Abdelsalam et al. [24] compared in-line blades with fins and concluded that the first was better. In its turn, Jonsson and Moshfegh [25] tested different heat sinks models considering Reynolds numbers in a range between 3350 and 13,400 using ANSYS Fluent software and concluded that it is not favorable to use pin heat sinks at higher Reynolds numbers.

When choosing a fin heat sink, it is typical to choose the conventional rectangular fins. Still, other forms of fins have been tested, such as triangular or trapezoidal fins [26,27], with fillets [28] or holes. Jaffal [29] analysed the thermal performance of different fin heat sinks geometries, via experimental and computational studies, at a certain heat flux interval. It was found that the heat transfer coefficient is dependent on heat flux and that the heat sink with perforated blades showed the best thermal performance. Through simulations in ANSYS software, Ibrahim et al. [30] investigated the effect of perforation geometry (circular, rectangular, and triangular) on the heat transfer of perforated fin heat sinks, under different boundary conditions. In all cases, these perforations increased the heat transfer coefficient and decreased heat sink temperature, regardless of perforation geometry. Tijani and Jaffri [31] also studied the effect of circular perforations on pins or fins heat sinks under forced convection. Inlet velocity and heat flux were constant and perforated pins or fins had the highest heat transfer coefficients, improving thermal efficiency up to 4% compared to solid pins or fins.

When choosing a pin heat sink, several authors agree that square pins are not a good choice [32,33,34] and that pressure drop is higher when pins arrangement is staggered [25,35,36,37]. However, there is no agreement regarding the best pins shape. Under forced convection, either circular [33,38], elliptical [32,39,40,41,42], dropform [43,44], and rhombus [45,46] can be advantageous. Gururatana and Li [40] compared elliptic and rectangular staggered pins with the same length-to-thickness ratio but with different hydraulic diameters. As boundary conditions, an inlet velocity of 6 m/s at 27 °C rendered a Reynolds number of 1192 (laminar flow). Through ANSYS Fluent simulations, they concluded that elliptic fins produced a higher heat transfer rate when the pressure drop is the same. Moreover, Zhou and Catton [39] investigated the thermal and hydraulic performance of different pin heat sinks with distinct pins shapes including square, circular, elliptic, and dropform. The elliptic pins had the best overall performance, regardless of inlet velocity and the ratio of pin widths to pins spacing. There are still other unusual shapes that can be a good choice [47,48]. Maji et al. [47] investigated the thermal performance of heat sinks with perforated circular pins. Results were taken for Reynolds numbers from 4700 to 44,500 and concluded that, up to a certain perforated area, perforated pins required lower pumping power than solid pins to reach the same thermal performance. Perforated pins were also investigated by other authors that also agree on their advantages [49,50].

The greatest fin or pin spacing is dependent on the air velocity, i.e., as the velocity increases, the fin spacing can decrease [3,51]. However, the dependence of heat transfer with fin or pin spacing is not clear. According to some authors [37,39,44], the heat transfer increases with increasing fin or pin density, i.e., with reduced spacing. Contrarily, according to other authors, greater heat transfer is obtained for the opposite [37,52,53].

### 1.2. Lattice Heat Sinks

As mentioned above, in the field of electronics cooling, where oversized heat sinks are inhibited by volume constraints, the use of additive manufacturing offers the ability to deliver components without the design restrictions of conventional manufacturing methods [48,54]. Lattice structures have showed high potential in increasing forced convection heat transfer. They consist of orderly unit cell arrangements, which can have different configurations such as hexagon, honeycomb, and pyramidal [55]. They have large surface area-to-volume ratios, are light, and promote tortuous fluid paths, promoting fluid mixing. Their advantage over metal foams are constant periodicity and homogeneity allowing optimization of the ligament configuration and diameter, better mechanical properties, and greater ease of production with emerging additive manufacturing technologies [54,55,56]. The study of the fluid flow through them has become popular in thermal management [55,56,57,58,59].

Although the lattice structure heat sink allows a high surface area to volume ratios, its performance may be limited by the absense of interaction between the cooling air and structure [7]. According to Ho et al. [55], pressure drop and Nusselt number of Rhombi-Octet lattice structures increased with decreasing unit cell size and the highest Nusselt number was obtained with the lattice structure with the smallest ligament width. The same conclusion was obtained by Son et al. [60].

Regarding the best unit cell topology for thermal management, there is no clear conclusion. For example, according to Yan et al. [61], the X-type lattice heat sink provides overall heat removal capacity up to two times higher than tetrahedral or the Kagome lattice heat sink. Its morphology resulted in a large scale spiral main flow that interacts with several secondary flows, causing three times higher pressure drop for a given Reynolds number. Still, superior heat transfer was achieved by the X-type lattice. The same conclusion was not made by Hyun and Torquato [62]. They suggested that Kagome structures have desirable heat-dissipation properties due to the large hexagonal holes through which fluid may flow, compared to triangular and hexagonal cells. More recently, Dixit et al. [63] concluded that octet topology dissipates more heat at the lowest Reynolds numbers while SC-BCC-truss outperforms other architectures as the fluid velocity increases.

## 2. Problem Description

### 2.1. CFD Methodology

**Figure 1:**
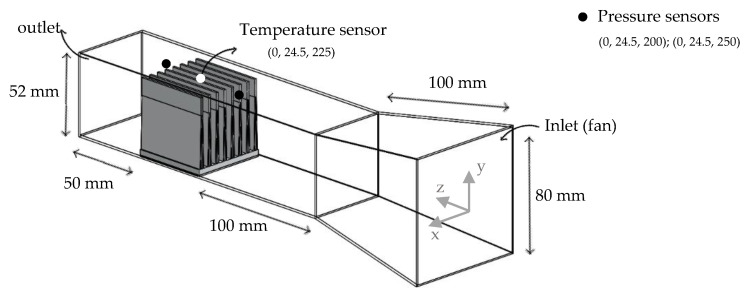
Computational domain.

As in other studies involving heat sinks, the main goal is to reach heat sink temperatures as low as possible, minimizing thermal resistance. The computational domain (Figure 1) includes a heat sink (main dimensions 50 × 50 × 50 mm^3^) and a wind tunnel, designed so that the total flow converges to the heat sink, avoiding the bypass phenomenon [25,55]. It was considered a fan diameter of 80 mm. The dimensions of the domain are a length of 300 mm and a converging width and height from 80 mm to 52 mm. Heat sink is placed inside the domain, 200 mm away from the inlet and 50 mm from the outlet.

The geometry was meshed using ANSYS Meshing by applying body sizing operation and, depending on the heat sink model under study, were considered tetrahedral or hexahedral elements. Element size varied in a range between 0.4 mm and 1.2 mm and the total number of elements was the one whose results converged, with minimal computational effort, i.e., sufficient to ensure mesh independence of the simulated results.

**Figure 2:**
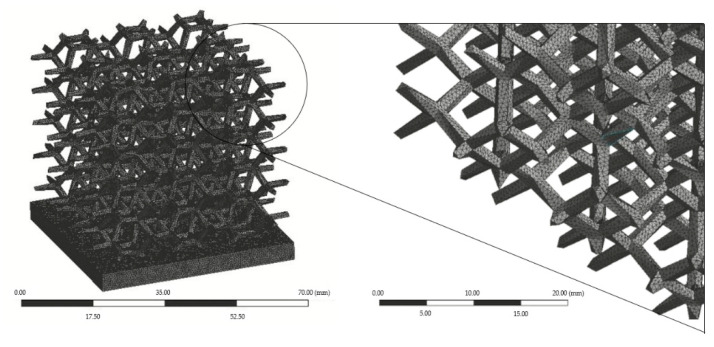
Lattice heat sink mesh.

Mesh geometry (Figure 2) was brought into Fluent, where solver settings were defined. This includes defining material properties, selecting appropriate physical models, prescribing operating and boundary conditions, and providing initial values. Table 1 includes the main properties of the materials adopted for each component: aluminium for the heat sink and ideal air as the fluid passing through the heat sink. The air flow was assumed incompressible with constant properties.

As initial values, the system was considered at a room temperature of 20 °C. The outlet vent condition was used for the outlet boundary and the wind tunnel wall considered adiabatic. The remaining boundary conditions (heat source temperature and inlet air velocity) were established according to each specific case.

During compute solution, the discretised conservation equations (Equations (4)–(6)) are solved iteratively until convergence, i.e., when changes in solution variables from one iteration to the next are negligible (residual response less than 10 × 10^−6^). As the most widely-used engineering turbulence model for industrial applications, standard *Ϗ*-Epsilon viscous model (Equations (6) and (7)) was selected. The pressure-velocity coupling was achieved through the *SIMPLE* scheme [64] and the *Least Squares Cell Based* gradients were choose as the spatial discretization scheme [65].

**Figure 3:**
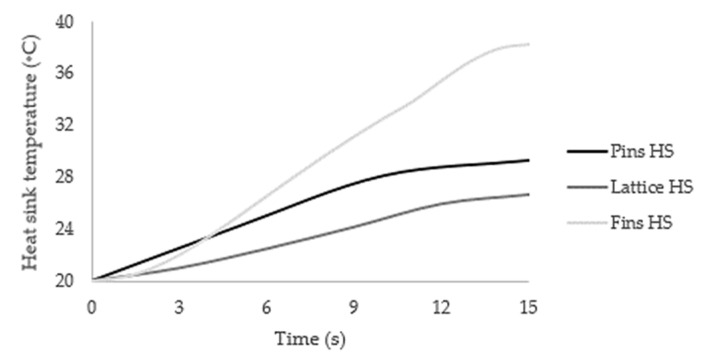
Temperature contours vs. time for each type of heat sink.

Pressure drop across the heat sink and its temperature were reported after 15 s on the respective sensors (Figure 1). The temperature sensor was located where, as general rule, the heat sink temperature was the minimum [66]. Considering a time step size of one second with 10 maximum iterations per time step, the 15 s were found to be a good balance between good results and computational effort (Figure 3) once, in most cases, the temperature reached the steady-state condition.

### 2.2. CFD Governing Equations

Computational Fluid Dynamics (CFD) consists of predicting fluid flow, heat, and mass transfer and related phenomena by solving numerically a set of governing mathematical equations. CFD analysis complements testing and experimentation by reducing total effort and cost required for experimentation and data acquisition. There are a number of commercial CFD software packages available for application in thermal design. One of them is ANSYS. Based on the finite volume method, ANSYS solvers discretised the domain into a finite set of control volumes in which general conservation equations for mass (Equation (2)), momentum (Equation (3)) and energy (Equation (4)) are solved [67].

(2) $$\nabla \cdot \left(\rho \vec{u}\right)=0$$

(3) $$\left(\vec{u}\cdot \nabla \right)\left(\rho \vec{u}\right)=\nabla P+\nabla \cdot \left(\mu \nabla \vec{u}\right)$$

(4) $$\vec{u}\cdot \nabla \left(\rho C_{p}T\right)=\nabla \cdot \left(k\nabla T_{i}\right)$$

Note that *ρ* and *μ* are the fluid density and viscosity and $\vec{u}$ is the velocity vector. The energy equation for solid regions [68] can be written as:

(5) $$k_{s}\nabla^{2}T=0$$

These equations are solved with the presumption that radiation heat transfer is negligible.

The standard *Ϗ*-Epsilon viscous model used in this study is a semi-empirical model based on model transport equations for the turbulence kinetic energy (*Ϗ*) and its dissipation rate (ε), obtained from the following transport equations:

(6) $$\frac{\partial }{\partial t}\left(\rho Ϗ\right)+\frac{\partial }{\partial x_{i}}\left(\rho Ϗu_{i}\right)=\frac{\partial }{\partial x_{j}}\left[\left(\mu +\frac{\mu_{t}}{\sigma_{Ϗ}}\right)\frac{\partial Ϗ}{\partial x_{j}}\right]+G_{Ϗ}+G_{b}-\rho \varepsilon -Y_{M}+S_{Ϗ}$$

(7) $$\frac{\partial }{\partial t}\left(\rho \varepsilon \right)+\frac{\partial }{\partial x_{i}}\left(\rho \varepsilon u_{i}\right)=\frac{\partial }{\partial x_{j}}\left[\left(\mu +\frac{\mu_{t}}{\sigma_{\varepsilon }}\right)\frac{\partial \varepsilon }{\partial x_{j}}\right]+C_{1\varepsilon }\frac{\varepsilon }{k}\left(G_{k}+C_{3\varepsilon }G_{b}\right)-C_{2\varepsilon \rho }\frac{\varepsilon^{2}}{k}+S_{\varepsilon }$$

In these equations, *G_Ϗ_* represents the generation of turbulence kinetic energy due to the mean velocity gradients, *G_b_* is the generation of turbulence kinetic energy due to buoyancy, *Y_M_* represents the contribution of the fluctuating dilatation in compressible turbulence to the overall dissipation rate, *C*_1_*_ε_, C*_2_*_ε_* and *C*_3_*_ε_* are constants, *σ_Ϗ_* and *σ*_ε_ are the turbulent Prandtl numbers for *Ϗ* and *ε*, respectively. *S_Ϗ_* and *S_ε_* are user-defined source terms. The *Ϗ*-epsilon model assumes that the flow is fully turbulent, and the effects of molecular viscosity are negligible [65,69].

### 2.3. Heat Sink Models

The statistical design of experiments (DOE) was used to design the sets of experiments run in this work for fins and pins heat sinks, in order to optimize the main geometric parameters (pins/fins diameter/thickness and pins/fins spacing).

As boundary conditions, the temperature of the heat source (at the bottom of the heat sink) varied between 80 °C and 100 °C, with 10 °C intervals, and inlet air velocity took the values 0.7 m/s, 2.1 m/s and 3.5 m/s (at 20 °C), leading to Reynolds numbers from 2500 (laminar-turbulent transition) to 12,500 (very turbulent flow).

For fins heat sinks, DOE analyses were performed using fin thickness, fin spacing, inlet velocity, and heat source temperature as factors and pressure drop and heat sink temperature after 15 s as responses. Each factor was considered with three levels (Table 2) and an L9 matrix was constructed.

For pins heat sinks, the arrangement factor (in-line or staggered) was added to compose an L18 DOE matrix. Level and factors are shown in Table 3.

**Figure 4:**
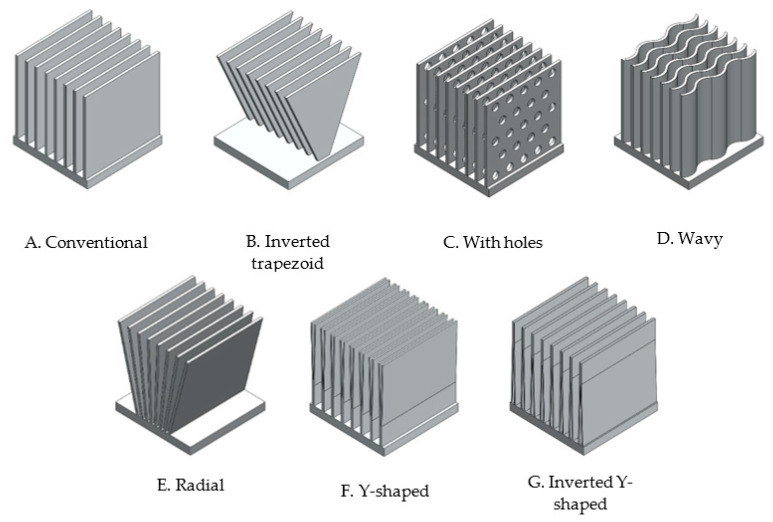
Different fins heat sink models.

**Figure 5:**
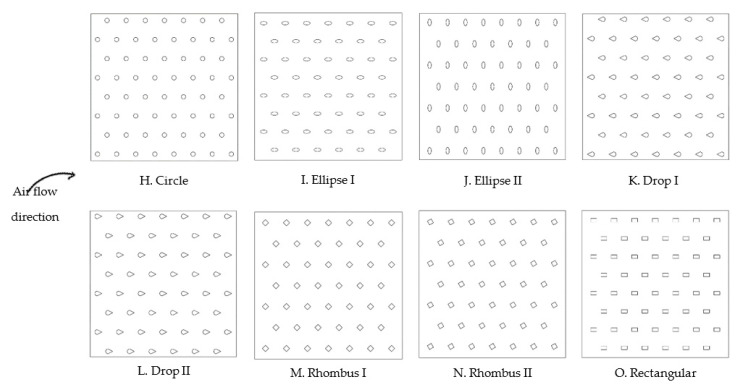
Different pins shapes under study.

Based on DOE results, the shape of fins or pins was varied according to some previous studies that showed good results [32,38,39,40,41,42,43,44,45,46,70] considering the same boundary parameters. For fins heat sinks (Figure 4), the same number of fins was considered. For pins heat sinks (Figure 5), the same hydraulic diameter and pin spacing was maintained.

**Figure 6:**
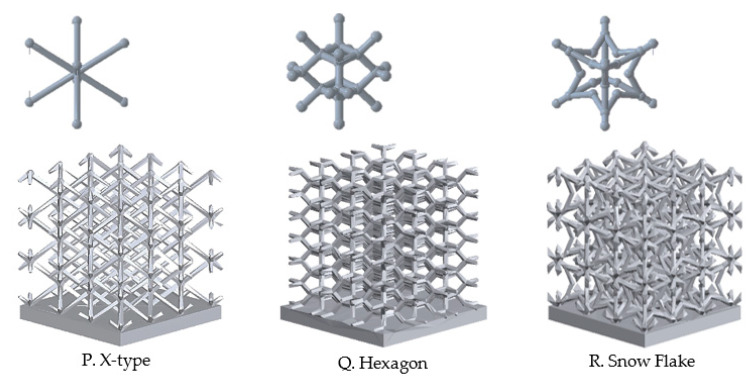
Different lattice heat sinks models.

In order to confirm the advantages of additive manufacturing for thermal management components, lattice sinks with X, Hexagon, and Snow Flake (Figure 6) unit cells were studied. Cell size and thickness were fixed as well as boundary conditions.

## 3. Results and Discussion

### 3.1. Fins Heat Sinks

**Figure 7:**
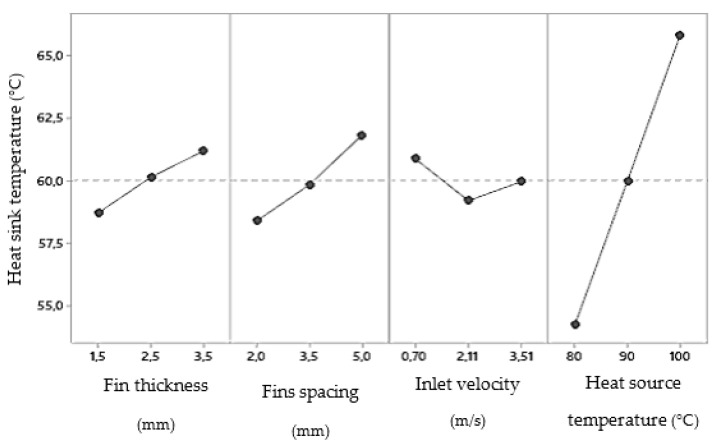

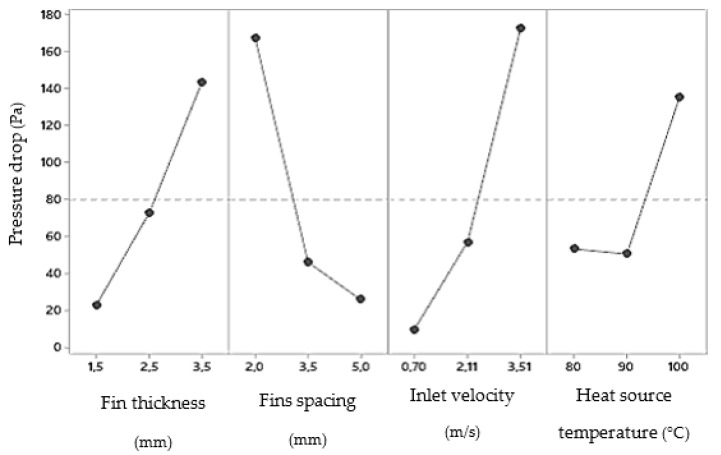
Main effects for heat sink temperature (**top**) and pressure drop (**bottom**) for fins heat sinks.

Using analysis of variance (ANOVA), the effects of variables and their interactions on each response were determined. For fins heat sinks, it was found that, either for a lower heat sink temperature (Figure 7, top) or for a lower pressure drop (Figure 7, bottom), the thickness of the fins should be as small as possible. The same was not true for the spacing. On the one hand, if it is as small as possible, it increases the density of the fins and therefore causes better thermal efficiency. On the other hand, the smaller the spacing, the higher pressure drops. In agreement between both parameters, and since the influence is much higher for the pressure drop, the bigger fins spacing (5 mm) was considered for further studies related to the shape of the fins (Figure 4).

Regarding boundary conditions, as expected, the effect of inlet velocity on pressure drop and heat source temperature on heat sink temperature after 15 s was linear. However, an inlet velocity of 2.11 m/s (Re = 7500) caused a minimum heat sink temperature and a heat source temperature of 90 °C caused a minimum pressure drop. These conclusions are related with the geometric parameters (number of fins and fins spacing), according to Table 4 and Table 5, respectively.

As boundary conditions, an inlet velocity of 2.11 m/s and a heat source temperature of 90 °C were considered for further studies with fins.

#### Fins Shape

In all fins heat sinks variants (Figure 4), the same number of fins (8) and boundary parameters were considered. Results for each model are shown in Table 6.

**Figure 8:**
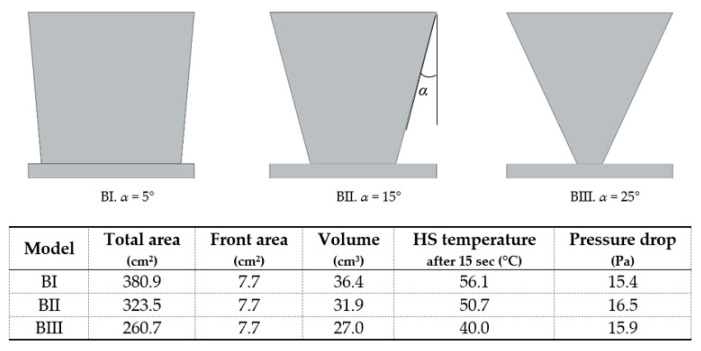
Model B variants.

It was found that the incorporation of fillets or chamfers did not bring any advantage, contrarily to all design variations shown in Figure 4 and Table 6. Fins with trapezoidal and inverted trapezoidal shape (Figure 4B) were considered, the latter being an advantageous option due to its wide part exposed to ambient air [27]. Moreover, the higher the inverted trapezoid angle, the better the performance (Figure 8).

**Figure 9:**
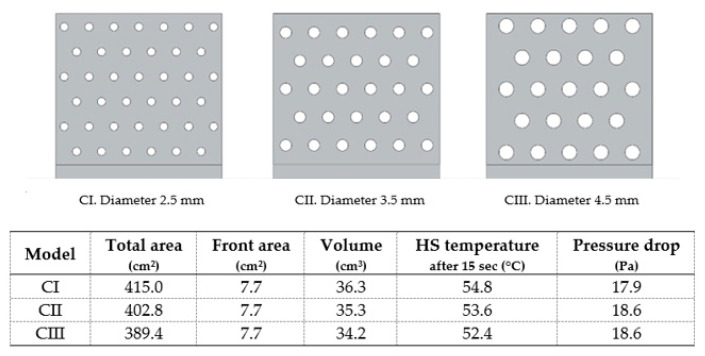
Model C variants.

The incorporation of holes in the fins (model C) has been studied by other authors [30,31] and both agreed that it was an advantageous approach due to the higher heat transfer coefficient. Furthermore, for larger holes diameter, maintaining holes spacing (5 mm) resulted in better heat sink performance (Figure 9).

**Figure 10:**
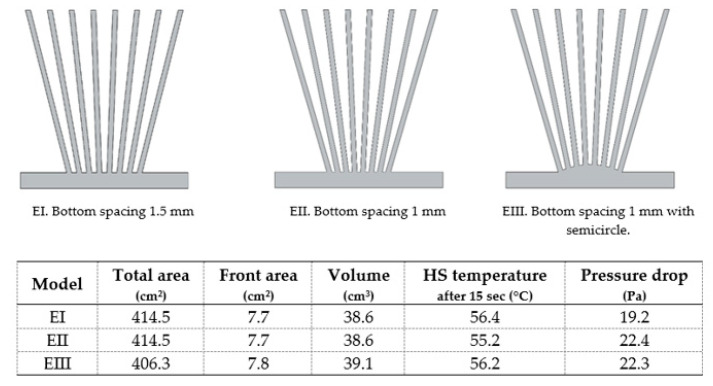
Model E variants.

Regarding model E, the lower the spacing between the fins at the bottom, keeping the top spacing equal to 5 mm, the better the heat sink performance (Figure 10, I and II). This happens because airflow towards radial fins tends to quicken as the gap between two consecutive fins reduces (EI to EII) [71]. These advantages of model E fins have also been confirmed experimentally by other authors [71,72]. However, the incorporation of more material in the heat sink base (Figure 10, III), close to the fins, did not bring any advantage.

**Figure 11:**
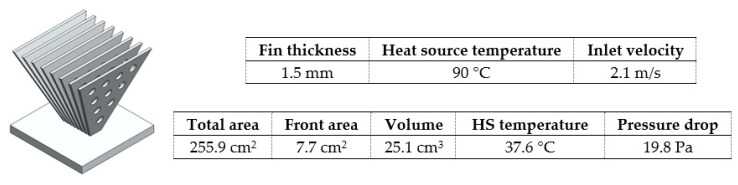
Best fin heat sink (among those studied).

With this, the properties of models B, C, and E have been combined and, among the studied, the heat sink design with the best performance was attained (Figure 11). Following some design rules for additive manufacturing, elliptical holes were considered instead of circular ones.

### 3.2. Pins Heat Sinks

**Figure 12:**
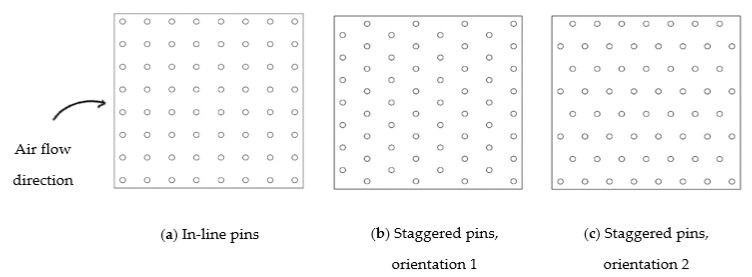
Results for the possible pin heat sinks arrangements and orientations.

For pins heat sinks, the arrangement factor was added: in-line or staggered pins (Figure 12). For staggered pins, there are two possible orientations (1 and 2). From orientation 1 to orientation 2, heat sink temperature increased by a maximum of 1.4% but pressure drop decreased by about 25%. Although the heat sink temperature after 15 s is the most pertinent response parameter, the decrease in pressure drop was much higher, causing a better performance for orientation 2 (Figure 12c). For this reason, the staggered arrangement was considered with orientation 2.

**Figure 13:**
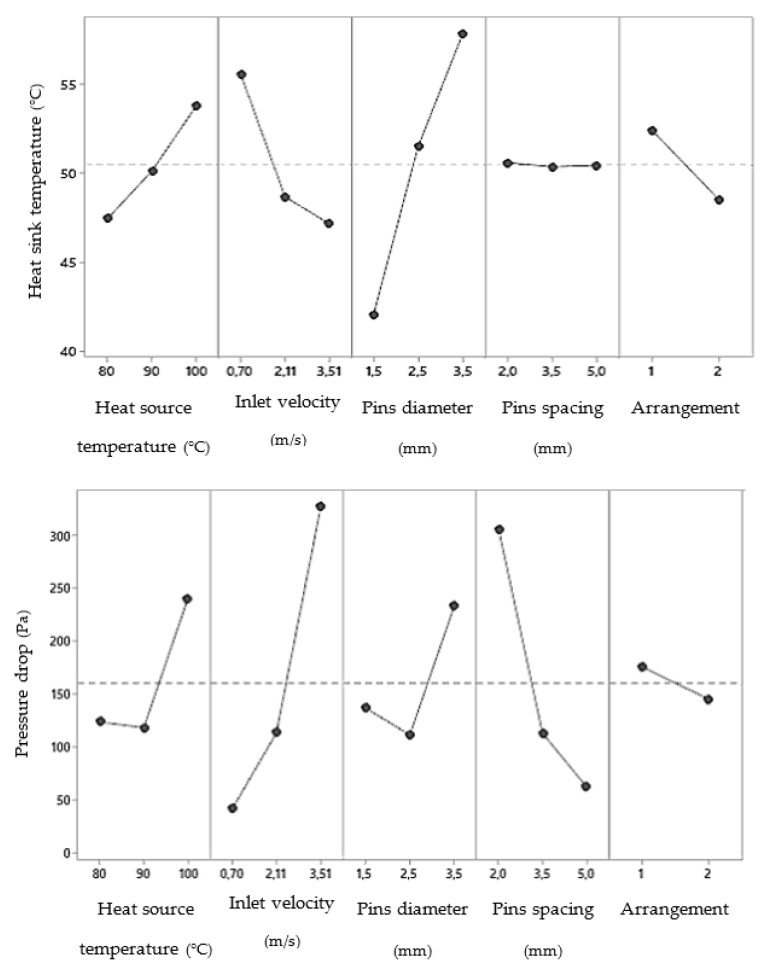
Main effects for heat sink temperature (**top**) and pressure drop (**bottom**) for pins heat sinks.

Taking this into account, it was found that, either for a lower heat sink temperature or for a lower pressure drop, the staggered arrangement (2) is more advantageous (Figure 13). Regarding pins diameter, for a lower heat sink temperature (Figure 13, top), it should be as small as possible. The same was not true for the pressure drop (Figure 13, bottom), where there is an ideal diameter of 2.5 mm. In agreement between both parameters, and since the heat sink temperature is the most relevant response parameter and the influence is more accentuated, the lower pins diameter (1.5 mm) will be considered for further studies related to the shape of the pins. With a smaller diameter, there is also a smaller building volume to be created by additive manufacturing.

Respecting pins spacing, if, on the one hand, there was no influence on the heat sink temperature, on the other hand, the influence on the pressure drop is quite noticeable. For this reason, the upper spacing (5 mm) was considered the best choice. These conclusions about geometric parameters are in agreement with the fin heat sinks.

As explained for fins heat sinks, a temperature of 90 °C caused a lower pressure drop (almost irrelevant) also due to some geometrical parameters (pins spacing and heat sink front area) given in Table 7. For this reason, this temperature was selected for further studies with pins. Regarding the inlet velocity, its influence was linear and as expected. Therefore, a velocity of 3.51 m/s was considered because it resulted in a lower heat sink temperature.

#### Pins Shape

In all pins shapes studies (Figure 5), the same hydraulic diameter (1.5 mm) and pin spacing (5 mm) were maintained, as well as boundary parameters. Results are shown in Table 8.

**Figure 14:**
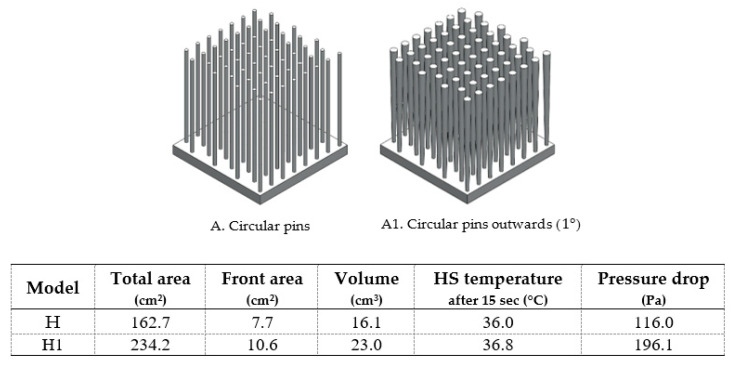
Comparison between circular pins (A) and circular pins outwards (A1).

Conventional heat sinks, with circular pins, continue to be the best choice among the many options of pin shapes, even though the pressure drop is high. For these pins, considering drawing outwards (Figure 14), only possible to be produced by additive manufacturing, was not advantageous—a higher temperature and a higher-pressure drop was obtained.

**Figure 15:**
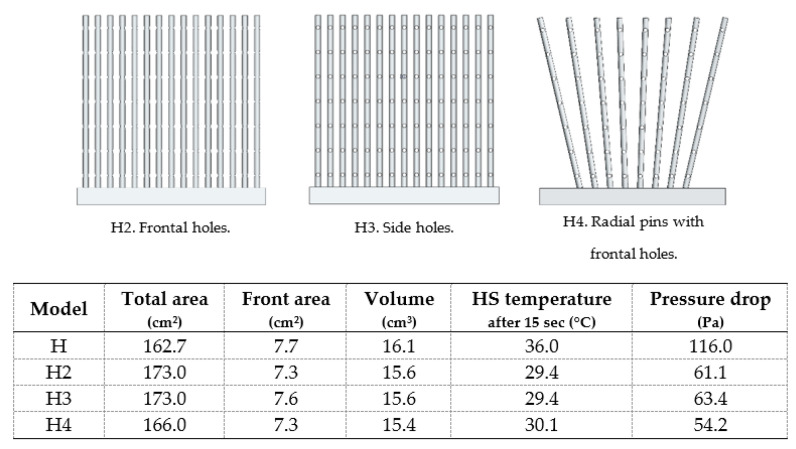
Pins heat sinks with frontal (H2) and side (H3) holes and radial pins heat sink with frontal holes (H4).

Figure 15 shows pins heat sinks with 1 mm frontal elliptical holes (H2) and side elliptical holes (H3) as well as a radial pins heat sink also with frontal elliptical holes (H4), spaced 5 mm apart. In all cases, holes were advantageous and the results were quite similar. For the same heat sink temperature obtained, the one with the lowest pressure drop was considered as the best option for pins heat sinks (H2).

For the case of ellipse pins (the second-best pins shape), differences were observed between maintaining the spacing (Model I) or maintaining the number of pins (Model I1), concerning the reference heat sink (Model H). According to Table 9, there were no significant differences, i.e., opting for ellipse pins, the choice should be Model I once it has a smaller building volume.

**Figure 16:**
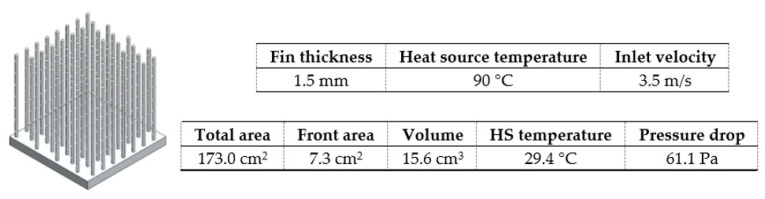
Best pin heat sink (among those studied).

Therefore, the best option contemplated circular pins in combination with frontal holes in their structure (Figure 16).

### 3.3. Lattice Heat Sinks

Lattice sinks with X, Hexagon, and Snow Flake (Figure 6) unit cells were studied. Cell size and thickness were fixed at 15 × 15 × 15 mm^3^ and 1.5 mm, respectively, as well as boundary conditions (heat source temperature of 90 °C and air inlet velocity of 3.5 m/s). The results are shown in Table 10.

Among the three models studied, the heat sink with hexagon unit cell showed the lowest temperature, despite the higher pressure drop. This heat sink has the middle total area to volume ratio. The advantages of this unit cell in thermal management applications were also confirmed by Gu et al. [73].

### 3.4. Fins vs. Pins vs. Lattice Heat Sink

**Figure 17:**
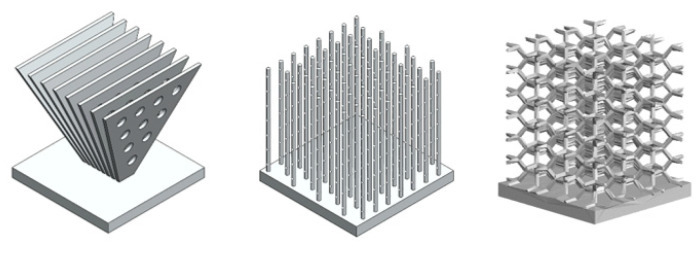
Fins, pins, and lattice heat sinks, respectively.

In this subchapter, it is intended to directly compare the best fins, pins, and blades heat sinks (Figure 17), under the same geometric and boundary parameters.

Table 11 shows that, under forced convection environments, better results were obtained for the lattice heat sink with hexagon unit cell. The pressure drop increased more than double for the same inlet velocity and Reynolds number, a consequence of the high frontal area. Even so, a decrease of about 28% and 9% in heat sink temperature was achieve, comparing with the best fins and pins heat sink, respectively. As inlet velocity and heat source temperature are kept constant during the experiments, the best heat sink is the one whose temperature is minimal, which, according to Equation (3), translates into lower thermal resistance.

Based on this study, there is a direct correlation between the total area to volume ratio and the heat sink performance. The lattice heat sink, the heat sink with the highest area to volume ratio and only possible to be produced by additive manufacturing, was considered the best option among the studied.

## 4. Conclusions

The thermal performance of heat sinks design under forced convection, varying geometric and boundary parameters (inlet velocity and heat source temperature), was conducted by computational fluid dynamics simulation with ANSYS Fluent.

Considering heat sinks with fins configuration, it was found out that the thickness of the fins should be as thin as possible and widely spaced. The optimal design is obtained by an agreement between both parameters. However, since the spacing has a greater impact on the pressure drop, the bigger fins spacing (5 mm) was considered for studies related to the shape of the fins. For this last iteration on the shape configuration, all geometric and boundary parameters were kept constant, varying only in heat sinks design. It was found that radial fins designed with an inverted trapezoidal shape and with holes have great advantages and the design (Figure 11) was the one with better performance.

A similar study was done for pins heat sinks. It was found that the best solution would be to consider a pin diameter of 1.5 mm with a spacing of 5 mm and with a staggered arrangement. By varying the shape of the pins, it was also found that the incorporation of holes in circular pins was beneficial for thermal performance.

Taking advantage of additive manufacturing freedom of design, three different lattice structures with the same cell size (15 × 15 × 15mm^3^) and thickness of 1.5 mm but different cell topology were compared. Among X, Hexagon, and Snow Flake unit cells, it was found that the Hexagon unit cell exhibited the best performance.

Through a direct comparison of the thermal efficiency of three heat sinks (the best fins, pins and lattice heat sinks), under the same boundary conditions, it was concluded that, under forced convection environments, a lattice heat sink with a hexagon unit cell is the optimal choice. These results validated how advantageous additive manufacturing can be for components that require thermal management, such as electronic devices. Even so, more studies should be treated to reduce pressure drop and optimize the lattice heat sink in terms of cell size and thickness.

## Figures and Tables

**Table 1:** Main properties of the materials considered for each component.

Property	Aluminium (Heat Sink)	Air (Fluid Domain)
Density (kg·m^3^)	2719	1.225
Specific heat (J·kg^−1^·°C^−1^)	871	1006.43
Thermal conductivity (W·m^−1^·°C^−1^)	202.4	0.0242
Viscosity (kg·m^−1^·s^−1^)	-	1.7894 × 10^−5^

**Table 2:** Levels and factors for DOE matrix for fins heat sinks.

Level	Fin Thickness (mm)	Fin Spacing (mm)	Inlet Velocity (m/s)	Heat Source Temperature (°C)
1	1.5	2.0	0.7	80
2	2.5	3.5	2.1	90
3	3.5	5.0	3.5	100

**Table 3:** Levels and factors for DOE matrix for pins heat sinks.

Level	Pin Diameter (mm)	Pins Spacing (mm)	Inlet Velocity(m/s)	Heat Source Temperature (°C)	Arrangement
1	1.5	2.0	0.7	80	In-line (1)
2	2.5	3.5	2.1	90	Staggered (2)
3	3.5	5.0	3.5	100	-

**Table 4:** Correlation between heat sink temperature and geometrical parameters.

Simulation	Number of Fins	Heat Source Temperature (°C)	Velocity (m/s)	HS Temperature after 15 s (°C)
#1	14	80	0.7	52.2
#2	6	80	2.11	56.4
#3	8	80	3.51	54.1
#4	7	90	0.7	61.9
#5	11	90	2.11	57.7
#6	8	90	3.51	60.5
#7	7	100	0.7	68.6
#8	10	100	2.11	63.5
#9	9	100	3.51	65.3

**Table 5:** Correlation between pressure drop and DOE factors.

Simulation	Spacing (mm)	Heat Source Temperature (°C)	Velocity (m/s)	Pressure Drop (Pa)
#1	2	80	0.7	13.8
#2	3.5	90	0.7	10.5
#3	5	100	0.7	4.4
#4	5	80	2.11	40.3
#5	2	90	2.11	108.4
#6	3.5	100	2.11	22.3
#7	3.5	80	3.51	105.1
#8	5	90	3.51	32.6
#9	2	100	3.51	380.1

**Table 6:** Temperature and pressure drop for each fin heat sink model.

Model	Total Area (cm^2^)	Front Area (cm^2^)	Volume (cm^3^)	HS Temperature after 15 sec (°C)	Pressure Drop (Pa)
A	408.9	7.7	38.6	60.6	13.4
B	260.7	7.7	27.0	40.0	30.6
C	389.4	7.7	34.2	52.4	25.1
D	397.3	20.2	35.1	53.9	52.6
E	414.5	7.7	38.6	55.2	22.4
F	682.1	7.7	38.6	52.0	31.5
G	682.1	7.7	38.6	54.5	24.5

**Table 7:** Correlation between pressure drop and geometrical parameters for an inlet air velocity of 3.51 m/s.

Arrangement	Simulation	Spacing (mm)	Front Area (mm^2^)	Heat Source Temperature (°C)	Pressure Drop (Pa)
In line	#1	3.5	13.4	80	289.7
	#2	5	10.2	90	167.1
	#3	2	16.6	100	776.9
Staggered	#4	3.5	11.4	80	170.7
	#5	5	7.7	90	116.0
	#6	2	11.8	100	441.1

**Table 8:** Temperature and pressure drop for each pin heat sink model.

Model	Nr of Pins	Total Area (cm^2^)	Front Area (cm^2^)	Volume (cm^3^)	HS Temperature after 15 s (°C)	Pressure Drop (Pa)
H	60	162.7	7.7	16.1	36.0	116.0
I	52	165.8	6.4	16.2	38.4	57.2
J	53	168.4	9.6	16.3	38.0	203.8
K	52	178.1	8.1	16.7	39.1	60.4
L	52	178.1	8.1	16.7	42.9	95.1
M	46	159.6	9.0	16.0	50.4	96.9
N	46	159.6	9.0	16.0	44.1	91.5
O	52	185.4	6.6	16.9	49.3	56.2

**Table 9:** Temperature and pressure drop for each pin heat sink model.

Model	Nr of Pins	Total Area (cm^2^)	Front Area (cm^2^)	Volume (cm^3^)	HS Temperature after 15 s (°C)	Pressure Drop (Pa)
H	60	162.7	7.7	16.1	36.0	116.0
I	52	165.8	6.4	16.2	38.4	57.2
I1	60	186.1	6.4	16.9	38.3	59.1
J	53	168.4	9.6	16.3	38.0	203.8

**Table 10:** Temperature and pressure drop for each pin heat sink model.

Model	Total Area (cm^2^)	Front Area (cm^2^)	Volume (cm^3^)	HS Temperature after 15 s (°C)	Pressure Drop (Pa)
X-type	187.9	31.3	16.8	39.2	108.1
Hexagon	243.7	41.1	19.2	26.7	161.2
Snow Flake	283.9	41.0	20.6	33.7	137.2

**Table 11:** Direct comparison between heat sinks with fins, pins, and blades (Re = 12,500).

Model	Total Are (cm^2^)	Front Area (cm^2^)	Volume (cm^3^)	Total Area to Volume Ratio	HS Temperature after 15 s (°C)	Pressure Drop (Pa)
Fins	255.9	7.7	25.1	10.2	36.9	50.2
Pins	173.0	7.3	15.6	11.1	29.4	61.1
Lattice	243.7	41.1	19.2	12.7	26.7	161.2

## Data Availability

Not applicable.

## Nomenclature

*q*	Heat Flux Density (W·m^2^)
*ρ*	air density (kg·m^−e^)
$\frac{\partial T}{\partial n}$	thermal gradient in direction *n* (K·m^−1^)
*t*	time (s)
*A*	heat transfer area (m^2^)
$\vec{u}$	velocity vector (m/s)
*P*	air pressure (Pa)
*R*	thermal resistance (K·W^−1^)
*T_f_*	fluid temperature (K)
*μ*	air viscosity (kg·m^−1^·s^−1^)
*C_p_*	Specific Heat Capacity (J·kg^−1^·K^−1^)
*Ϗ*	turbulence kinetic energy (J·kg)
∆*T*	difference between the minimum temperature of the HS and the fluid temperature at the inlet (K)
*Q_heat_*	total heat applied at the base of the HS (W)
*h*	convective heat transfer coefficient (W·m^−2^·K^−1^)
*k*	thermal conductivity (W·m^−1^·K^−1^)
*T_s_*	HS surface temperature (K)
*T_i_*	inlet air temperature (K)
*σ_Ϗ_*	turbulent Prandtl number for *Ϗ*
*σ_ε_*	turbulent Prandtl numbers for ε
**Acronyms**	
CFD	Computational Fluid Dynamics
DOE	Design of experiments
HS	Heat sink
